# *Neisseria gonorrhoeae* co-infection exacerbates vaginal HIV shedding without affecting systemic viral loads in human CD34^+^ engrafted mice

**DOI:** 10.1371/journal.pone.0191672

**Published:** 2018-01-23

**Authors:** Stacey X. Xu, Danila Leontyev, Rupert Kaul, Scott D. Gray-Owen

**Affiliations:** 1 Department of Molecular Genetics, University of Toronto, Toronto, Ontario, Canada; 2 Department of Medicine, University of Toronto, Toronto, Ontario, Canada; 3 University Health Network, University of Toronto, Toronto, Ontario, Canada; University of Pittsburgh, UNITED STATES

## Abstract

HIV synergy with sexually transmitted co-infections is well-documented in the clinic. Co-infection with *Neisseria gonorrhoeae* in particular, increases genital HIV shedding and mucosal transmission. However, no animal model of co-infection currently exists to directly explore this relationship or to bridge the gap in understanding between clinical and *in vitro* studies of this interaction. This study aims to test the feasibility of using a humanized mouse model to overcome this barrier. Combining recent *in vivo* modelling advancements in both HIV and gonococcal research, we developed a co-infection model by engrafting immunodeficient NSG mice with human CD34^+^ hematopoietic stem cells to generate humanized mice that permit both systemic HIV infection and genital *N*. *gonorrhoeae* infection. Systemic plasma and vaginal lavage titres of HIV were measured in order to assess the impact of gonococcal challenge on viral plasma titres and genital shedding. Engrafted mice showed human CD45^+^ leukocyte repopulation in blood and mucosal tissues. Systemic HIV challenge resulted in 10^4^−10^5^ copies/mL of viral RNA in blood by week 4 post-infection, as well as vaginal shedding of virus. Subsequent gonococcal challenge resulted in unchanged plasma HIV levels but higher viral shedding in the genital tract, which reflects published clinical observations. Thus, human CD34^+^ stem cell-transplanted NSG mice represent an experimentally tractable animal model in which to study HIV shedding during gonococcal co-infection, allowing dissection of molecular and immunological interactions between these pathogens, and providing a platform to assess future therapeutics aimed at reducing HIV transmission.

## Introduction

The synergy between sexually transmitted infections (STIs) and human immunodeficiency virus (HIV) is well-recognized [1]. While meta-analyses show varied and sometimes negligible effects of STI treatment on host HIV pathogenesis [2,3], these studies often do not delineate between individual STIs, which could mask pathogen-specific effects. For instance, HIV-positive men with urethritis, and gonococcal urethritis in particular, exhibit higher levels of seminal HIV shedding [4–6]. Moreover, HIV-positive women treated for gonococcal cervicitis displayed decreased cervical viral shedding [7]. These clinical observations suggest that there is specific HIV-gonococcal synergy, and molecular studies provide compelling evidence that gonococcal-specific factors, rather than general inflammation, drive the interaction with HIV. Confoundingly, studies that aimed to elucidate the nature of this HIV-gonococcal synergy revealed that co-infection by *Neisseria gonorrhoeae* can suppress, as well as enhance, HIV infection through varied mechanisms. Various gonococcal components can affect HIV infection in different ways: while *N*. *gonorrhoeae* lipooligosaccharide drives a potent host immune response capable of suppressing HIV infection [8], *Neisseria* also shed the metabolite heptose-1,7-bisphosphate, which stimulates HIV long terminal repeat-driven expression to increase viral replication and drive the virus from latency [9,10]. HIV-gonococcal interactions also vary depending on the type of immune cell examined. For instance, *N*. *gonorrhoeae* stimulates TLR2 activation to promote HIV infection in resting primary T cells [11] and dendritic cells [12], yet prevents DC-mediated priming of HIV-specific memory responses [13]. However, the interferon-α response by *N*. *gonorrhoeae*-infected primary T cells opposes viral replication [14]. Clinically, HIV-specific CD8^+^ responses are noticeably impacted by gonococcal co-infection, but different outcomes seem to depend upon HIV status at the time of co-infection [15,16]. Clearly these studies reveal a complicated picture, which prevents disparate *in vitro* experimental and clinical observations from being integrated into a model explaining the impact of HIV/*N*. *gonorrhoeae* co-infection.

Since *N*. *gonorrhoeae* and HIV are both human-specific pathogens with different requirements for establishing infection, a physiologically relevant experimental model suitable for co-infection studies remains undeveloped. HIV infection is supported in ‘humanized’ mouse models, whereby immunodeficient mice are transplanted with human hematopoietic stem cells (HSC) from bone marrow, cord blood and/or fetal tissues, which differentiate into mature leukocytes including target CD4^+^ cells [17]. Advancements in the modelling of *N*. *gonorrhoeae* infection and disease [18,19] suggest that it may now be feasible to combine these models to study co-infection in a tractable model. Here we describe studies to establish the feasibility and provide proof-of-principle for a HIV/*N*. *gonorrhoeae* co-infection model, and report the effect of mucosal gonococcal infection on systemic HIV infection and genital shedding.

## Materials and methods

### Humanized mouse generation

Female NOD/*LtSz-scid/scidγc*^*null*^ (NSG) mice aged 4 weeks were purchased from Jackson Labs. At 5 weeks of age these mice (15–20g) were administered two 25 mg/kg doses (DMSO stock diluted with RPMI 1640) of the chemotherapeutic alkylating agent busulfan 24 hours apart via intraperitoneal (i.p.) injection, to deplete mouse bone marrow cells and permit engraftment of human cells [20]. Twenty-four hours following the last busulfan dose, 2 x 10^5^ CD34^+^ HSC from fetal liver or pooled cord blood thawed from frozen (Stem Cell Technologies) were injected via tail-vein. Peripheral blood engraftment was evaluated at week 18 post-transplant by flow cytometry. Antibodies used included anti-mouse CD45 (clone 30-F11) conjugated to PerCP-Cy5.5 (eBiosciences), anti-human CD45 (clone 2D1) conjugated to APC-Cy7 (BD), anti-human CD33 (clone WM53) conjugated to Alexa Fluor 700 (BD), anti-human CD3 (clone UCHT1) conjugated to PE-Cy7 (eBiosciences), anti-human CD4 (RPA-T4) conjugated to APC (BD), anti-human CD8β (clone 2ST8.5H7) conjugated to ECD (Beckman Coulter), anti-human TCRαβ (clone IP26) conjugated to FITC (eBioscience), anti-human CD19 (clone HIB19) conjugated to PE (eBioscience), anti-human CCR5 (clone 2D7/CCR5) conjugated to Brilliant Violet 421 (BD), and live/dead fixable aqua (Molecular Probes). For tissue engraftment studies, mice were euthanized with CO_2_ at 18 weeks post-engraftment. Tissues were minced with scissors and incubated with collagenase D (Roche) to dissociate into a single cell suspension for flow cytometry. Cells were acquired using a LSRFortessa (BD) and analyzed using FlowJo v10 (Treestar).

### Ethics and animal care

All animal procedures were approved by the Animal Ethics Review Committee at the University of Toronto, in strict accordance with provincial and federal ethical and legal requirements (protocol number 20011003). Mice were housed in a specific pathogen free facility with temperature and humidity regulation, light/dark cycles, housing enrichment, and provided with standard chow and facility-filtered water *ad libitum*. Mice were anesthetized with isoflurane for all procedures and monitored daily for clinical signs of sickness (graft-versus-host disease, weight loss, inactivity) for the duration of the experiment, beginning with myelablation-engraftment. Two mice were euthanized due to graft-versus-host complications prior to any infections. While the mice did not typically show signs of severe sickness after infections, 2 mice were euthanized over the course of 7 weeks following HIV infection. After vaginal and transcervical infections, 2 PBS-inoculated and 1 *N*. *gonorrhoeae*-infected mice were euthanized under anesthesia. All animals were humanely euthanized via exsanguination under anaesthesia according to clinical endpoint or at the experimental endpoint, with every effort to minimize suffering.

### HIV infection and viral titres

HIV-1 BaL (TCID = 10 000) was injected i.p. to establish infection in 18 repopulated mice. Blood samples were taken bi-weekly via saphenous bleed in EDTA-coated tubes and 20 μL plasma collected. Mucosal lavages were taken weekly or as indicated by pipetting 30 μL of PBS in and out of the vagina gently. Each sample was diluted (30 μL into 1 mL of deionized distilled water) and analyzed for viral RNA using the m2000 RealTime HIV-1 Viral Load Assay (Abbott Molecular).

### *N*. *gonorrhoeae* infections

For vaginal infections, mice were pre-treated starting two days pre-infection with i.p. injections of streptomycin sulfate (2.4 mg) and vancomycin hydrochloride (0.6 mg) diluted in PBS, and trimethoprim sulfate (0.04 g/L) in drinking water to suppress vaginal microbiota as previously described [18]. Concurrent hormone injections of 0.5 mg water-soluble 17β-estradiol (Sigma) dissolved in PBS were also administered every other day, starting two days before *N*. *gonorrhoeae* infection, to synchronize the mice in estrus [21]. Low-passage clinical isolates of *N*. *gonorrhoeae* [19] were lawn-streaked on GC agar (Difco) supplemented with IsoVitalex and VCNT antibiotics (BD) overnight, washed and prepared in PBS^++^ (Life Technologies), and either 10 μL of PBS^++^ or 1 × 10^8^ CFU *N*. *gonorrhoeae* in 10 μL of PBS^++^ was introduced vaginally with a pipette. One month after vaginal infection, mice were transcervically inoculated with either 1 × 10^8^ CFU *N*. *gonorrhoeae* or 20 μL of PBS using a blunt 25-gauge needle (Sai Infusion Technologies), as previously described [19]. Five days prior to transcervical infection, mice were subcutaneously injected with 2 mg medroxyprogesterone acetate (Pfizer) to synchronize mice to diestrus stage.

### Statistical analyses

All statistical analyses were performed using Prism v.7 (GraphPad) or SPSS v. 24 (IBM). Paired changes over time were analyzed using Wilcoxon rank-test, and Pearson’s chi-square test was performed for categorical variables to evaluate if mice co-infected with *N*. *gonorrhoeae* shed HIV vaginally or not. P values equal to or less than 0.05 were considered to be statistically significant.

## Results

### The mouse vaginal mucosa becomes populated by human CD45^+^CD4^+^ cells

**Fig 1:**
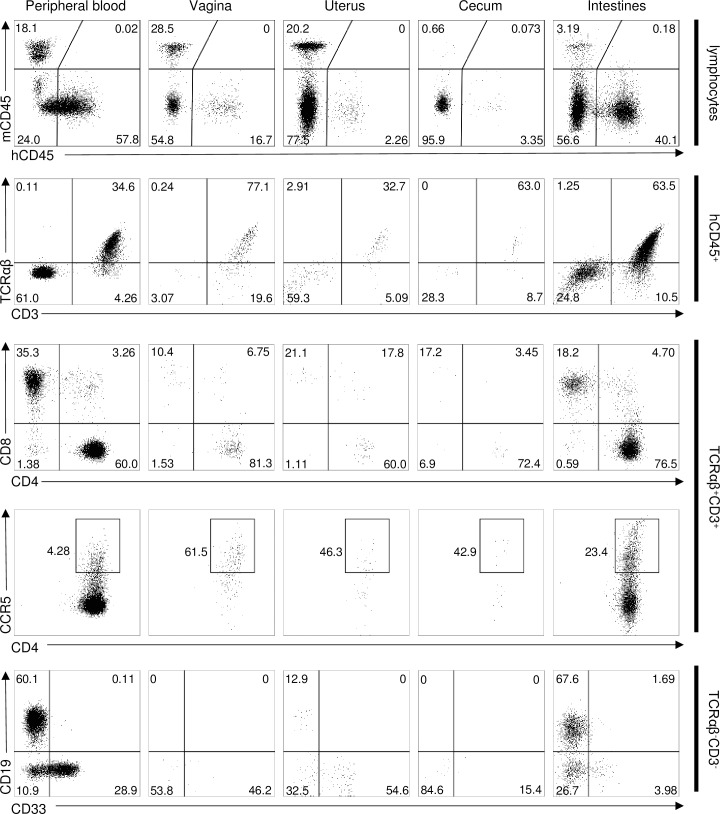
Humanized CD34^+^ HSC-transplanted NSG mice exhibit CCR5^+^CD4^+^ T cell repopulation in peripheral blood and mucosal tissues. Blood and mucosal tissues from human cord blood-derived CD34^+^ HSC-transplanted NSG mice were analyzed by flow cytometry 18 weeks post-engraftment. Cells were analyzed via doublet-exclusion, viability staining and lymphocyte-gating. Parent gate is indicated on the right.

Using flow cytometry, we observed engraftment of NSG mice with high levels of human CD45^+^ cells in peripheral blood, including CD4^+^ T cells and a subset of these expressing the HIV co-receptor CCR5, 18 weeks after administering CD34^+^ HSC from either cord blood (Fig 1) or fetal liver (S1 Fig). Analysis of the mucosal tissues also revealed that human CD45^+^ leukocytes, including CD4^+^ and CD4^+^CCR5^+^ T cells which are the primary host cells for HIV, also populate mucosal tissues in the transplanted animals (Fig 1).

### Humanized mice exhibit genital HIV shedding

**Fig 2:**
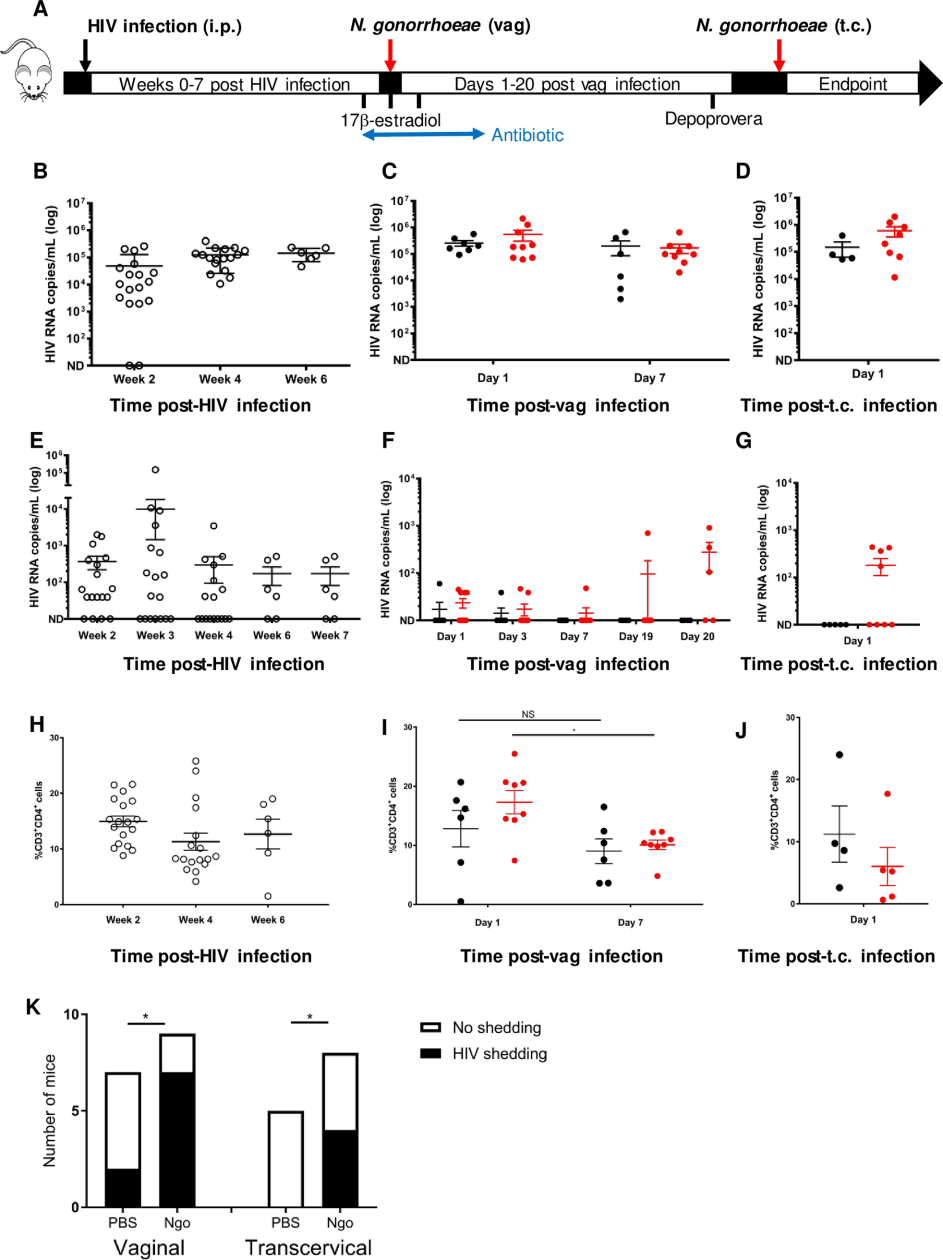
HIV titres and CD4^+^ T helper cells in plasma and vaginal lavage with and without *N*. *gonorrhoeae* co-infection. A) Schematic of experimental infection procedure following 18-week engraftment period for CD34^+^ HSC-transplanted NSG mice, including hormone delivery and antibiotic schedule, as described in Materials and Methods. HIV titres (RNA copies/mL) following i.p. infection with TCID 10 000 HIV-1 BaL in B) plasma and E) vaginal lavage (Open circle = HIV only). HIV titres following vaginal (vag) and transcervical (t.c.) inoculation with *N*. *gonorrhoeae* (red circle) or PBS (black circle) in plasma (C,D) and vaginal lavages (F,G). Limit of detection ≤40 copies HIV RNA per 20 μL of plasma or 30 μL of vaginal lavage; not detectable (ND). CD4^+^ T helper levels in blood (H-J) as measured by flow cytometry (gated on live hCD45^+^ cells) and analyzed for paired changes using Wilcoxon rank-test with * denoting p≤0.05, no significance (NS). Each replicate denotes samples from one mouse and error bars denote the standard error of the mean. (K) Categorical variable analysis of whether or not *N*. *gonorrhoeae* (Ngo) infection resulted in vaginal HIV shedding with data from F and G, * denoting p≤0.05 as determined by Pearson’s chi-square test (two-tailed).

After establishing high levels of human CD45^+^ engraftment in peripheral blood (S1 Fig), 18 humanized mice were inoculated systemically with HIV in order to evaluate the effects of gonorrhea on established HIV infections. Bi-weekly blood sampling showed that all mice established systemic HIV infection by 4 weeks post-infection, although 6 mice required a second viral challenge (Fig 2B). The latter mice achieved similar plasma viral titres (data not shown). CD4^+^ T helper cell counts in blood were stable following HIV infection, with a transient although not statistically significant drop around week 4 (Fig 2H), indicative of successful HIV establishment in the host. Interestingly, weekly vaginal lavages revealed viral titres of up to 1.5 x 10^5^ copies/mL, peaking at week 3 and then declining thereafter. Not all mice had detectable levels of HIV in lavages at each time point, but every mouse had detectable HIV^+^ in at least one lavage prior to mucosal *N*. *gonorrhoeae* challenge (Fig 2E).

### *N*. *gonorrhoeae* infection enhances HIV shedding in the female genital tract (FGT) but does not affect plasma titres

After 4–7 weeks following HIV infection and establishment of systemic viral titres (see schematic in Fig 2A), these HIV-infected mice were administered β-estradiol to synchronize them in estrus phase of the reproductive cycle and antibiotics to suppress the vaginal microbiome. These mice were then randomized into two separate groups and then vaginally administered either PBS or *N*. *gonorrhoeae*. These groups did not differ in HIV titres of blood (Fig 2C), although CD4 T helper cells decreased 7 days following vaginal infection with *N*. *gonorrhoeae* compared to one day post-infection, which was not evident in PBS-treated mice (Fig 2I). HIV titres in vaginal lavages also did not initially appear to be different between groups, however an increase was observed 19–20 days after mice were infected with *N*. *gonorrhoeae* relative to the PBS only controls (Fig 2F). Using Pearson’s chi-square test, vaginal *N*. *gonorrhoeae* infection significantly increases the likelihood that a mouse will mucosally shed HIV (Fig 2K).

Previous work has established that vaginal *N*. *gonorrhoeae* infection results in relatively modest levels of inflammation, with neutrophil, cytokine and chemokine influx peaking around day 5 [22]. To consider how the immunopathology associated with pelvic inflammatory disease influenced viral shedding, we took advantage of an established protocol whereby *N*. *gonorrhoeae* are inoculated transcervically to allow direct access to the uterine horns. This infection elicits a rapid inflammatory cytokine response and leukocyte recruitment within 6 hours [19]. To accomplish this, the vaginally-infected mice were transcervically re-infected with *N*. *gonorrhoeae* 4 weeks after the primary infection, while the PBS-treated mice were sham (PBS) inoculated. Transcervical gonococcal infection led to higher vaginal shedding of HIV (Fig 2G and 2K), while plasma HIV levels again remained unchanged (Fig 2D). Notably, none of the mice that were transcervically administered PBS shed any virus, highlighting that the effect of gonococcal administration does not result from the transcervical inoculation procedure (Fig 2K). CD4 T cell levels were not significantly different between PBS and gonococcal-treated mice (Fig 2J), supporting a compartmentalization of the mucosal response to gonorrhea. When comparing the viral shedding after the two *N*. *gonorrhoeae* infection models, it is also notable that vaginal HIV emerged several weeks after lower genital tract infection but was observed within 24 hours of the gonococci being administered to the upper genital tract.

## Discussion

Prior to this study, successful mucosal repopulation by human cells in the FGT had been reported in CD34^+^ HSC-transplanted *Rag2*^*null*^γ*c*^*null*^ mice based upon immunohistochemistry [23], but mucosal characterization has not otherwise been performed in CD34^+^ HSC ‘cell transplant-only’ mouse models (i.e. without additional fetal tissue implantation) such as the NSG mice. Here we demonstrate that human CD34^+^ HSC-transplanted NSG mice repopulate mucosal tissues including the FGT, with high levels of CD4^+^CCR5^+^ cells in line with previous observations in both simians and humans [24–26]. Importantly, these mice exhibit HIV mucosal shedding that was previously unappreciated. Assessment of genital shedding in this model can, therefore, be used as a readout for pre-clinical testing of microbicides and topical anti-retroviral therapies, as well as genital infection. Although limitations do exist in this model, such as the reported superiority of mucosal HIV challenge and T cell development in human thymus tissues present in more ‘humanized’ models, such as bone marrow-liver-thymus (BLT) mice [17,27], we suggest that using CD34^+^ HSC-transplant NSG mice represents a more readily available alternative for some HIV shedding studies.

By coupling this HIV model with *N*. *gonorrhoeae* infections, we were able to establish, for the first time that we are aware, HIV-gonococcal co-infection within a model host. Secondary infection with *N*. *gonorrhoeae* in the FGT of HIV-infected mice revealed an interesting disconnect between the systemic and mucosal compartments, where mucosal HIV titres increased compared to the control group but plasma HIV levels were unaffected. CD4 levels remained relatively stable even as systemic HIV levels remained high, although we observed a decrease 7 days following the vaginal introduction of *N*. *gonorrhoeae* that was not evident in the PBS controls.

The role of hormones and the microbiota is well-recognized to impact HIV replication. Since the PBS control mice were treated with the same antibiotic and hormone regimens as those infected with *N*. *gonorrhoeae*, the effects of varying microbiota and hormones on HIV can be ruled out [28,29]. Overall, our data suggests that HIV/*N*. *gonorrhoeae* interactions that promote viral shedding occurs at the local mucosal level. This mucosal-specific phenomenon has been previously observed in HIV^+^ men, where concurrent gonococcal urethritis was associated with higher HIV in semen but not plasma [4,5], supporting the physiological relevance of this co-infection model.

In humans, plasma HIV loads generally correlate with genital HIV levels and the risk of HIV transmission [30], which is why antiretroviral therapy (ART) has been so successful in reducing transmission risk [31]. However, despite plasma titres reaching undetectable levels, ART is not always able to completely suppress mucosal HIV and this can partially be attributed co-infecting STIs [32]. Future studies dissecting the mechanism of enhanced viral shedding (free virus vs. infected cells), how the host immune response to *N*. *gonorrhoeae* affects recruitment of infected cells, viral replication, *de novo* infections, latency and persistent infections are all important avenues for future exploration using this novel mouse model.

## Conclusion

Humanized mouse models represent an exciting new approach to study infection and disease by human-specific pathogens such as HIV and gonorrhea. While the majority of HIV and gonococcal transmission occurs through sexual contact, HIV shedding and interactions with STIs at mucous membranes has remained unexplored due to the lack of an appropriate animal model. Here we show that CD34^+^-transplanted NSG mice not only repopulate the FGT with human leukocytes, but also exhibit mucosal HIV shedding, which was previously unappreciated. In this study, we establish the first experimental HIV/*N*. *gonorrhoeae* animal co-infection using *N*. *gonorrhoeae* infection routes that either establish lower genital tract colonization or upper genital tract inflammatory disease. We observe that mucosal exposure to *N*. *gonorrhoeae* via either of these routes results in enhanced vaginal HIV shedding without any observable effect on systemic HIV titres, although the onset of HIV shedding is much more rapid upon the establishment of uterine infection. These findings reflect nicely what has been observed in humans, demonstrating the feasibility and clinical relevance of this animal model for studying mucosal responses and co-infection relationships. In the ongoing search for a cure, advancements in animal models offer hope for better understanding molecular and immunologic aspects of co-infection and for testing future interventions to reduce HIV transmission.

## Supporting information

S1 Fig Peripheral blood engraftment of fetal liver CD34+-transplanted NSG mice. Percentage of leukocytes in NSG mice (n = 18) 18 weeks post-engraftment with fetal liver CD34^+^ hematopoietic stem cells. Populations were gated based on doublet-exclusion, live/dead staining and lymphocytes on the basis of forward and side scatter. Each dot represents one animal and error bars represent standard error of the mean.
(TIF)

S1 File ARRIVE guidelines checklist. (PDF)
