# Supplementary figures and images for: Neisseria gonorrhoeae co-infection exacerbates vaginal HIV shedding without affecting systemic viral loads in human CD34+ engrafted mice
Source: PLoS One. 2018 Jan 23;13(1):e0191672. doi: 10.1371/journal.pone.0191672 (PMC5779692; doi:10.1371/journal.pone.0191672)

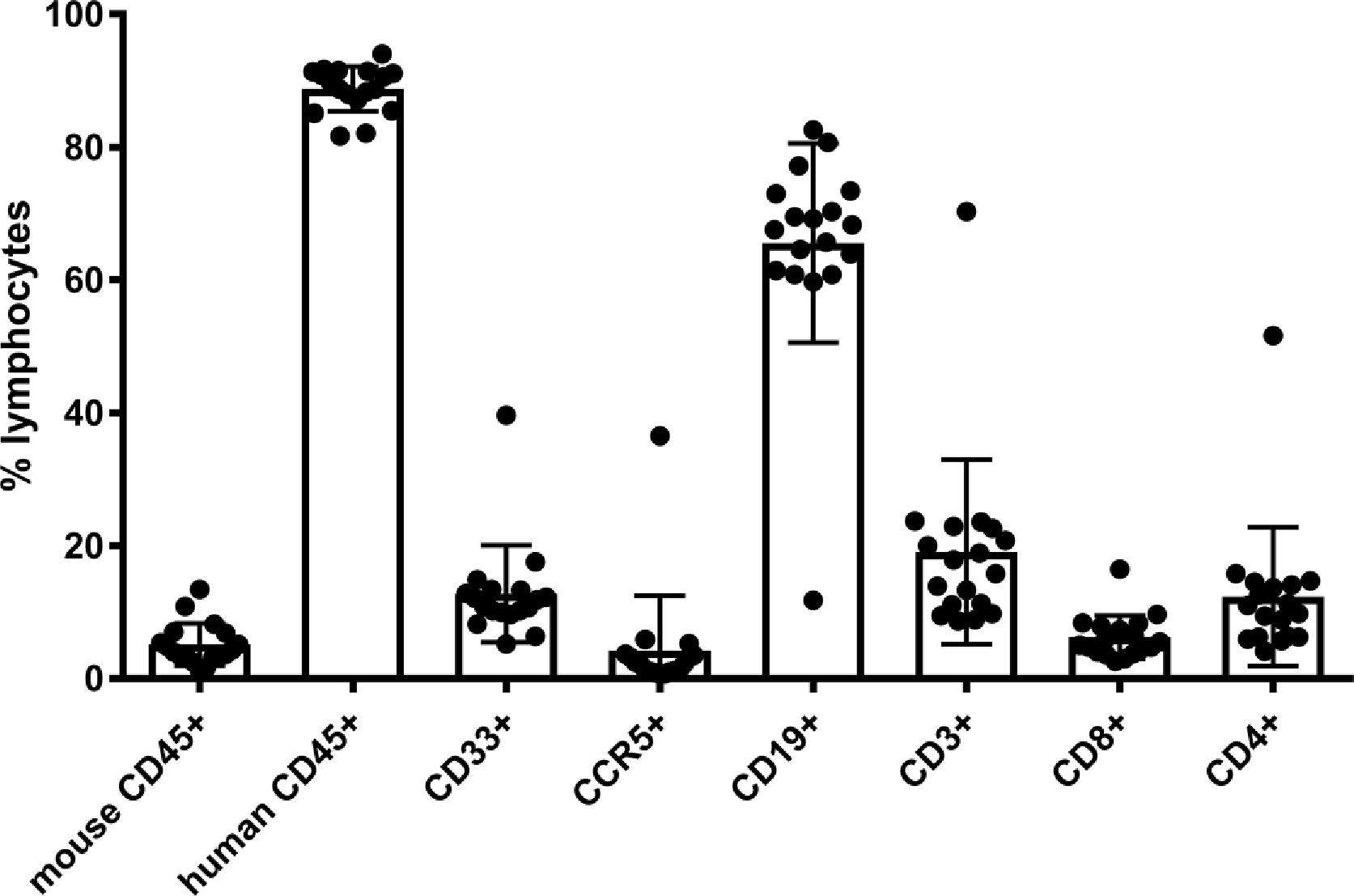

Supplement: S1 Fig — Percentage of leukocytes in NSG mice (n = 18) 18 weeks post-engraftment with fetal liver CD34+ hematopoietic stem cells. Populations were gated based on doublet-exclusion, live/dead staining and lymphocytes on the basis of forward and side scatter. Each dot represents one animal and error bars represent standard error of the mean. (TIF) [file pone.0191672.s001.tif]
